# The Accuracy of Digital Preoperative Templating in Primary Total Hip Replacements

**DOI:** 10.7759/cureus.43046

**Published:** 2023-08-06

**Authors:** Sameh F Girgis, Suraj Kohli, Georgios Kouklidis, Abdelfatah M Elsenosy, Omer Ahmed, Lawrence O'Hara, Michael Kent, Bassem Michael, Nedal Zeineh

**Affiliations:** 1 Trauma and Orthopaedics, University Hospitals Dorset, Bournemouth, GBR; 2 Trauma and Orthopaedics, Dorset County Hospital, Dorchester, GBR

**Keywords:** kingmark, digital planning, accuracy, total hip arthroplasty: tha, preoperative templating

## Abstract

Background​

Digital templating is an essential part of preoperative planning in elective total hip replacement (THR) surgery. The goals of templating are to predict femoral and acetabular implant sizes, to assess leg length, offset, and implant positioning. Templating markers such as the KingMark device (Brainlab, Munich, Germany) have been developed to improve the accuracy. Although templating is commonly used in many centres, there are challenges related to the accuracy of the process, such as true magnification ideal positioning of the pelvis and hips/body habit (obesity).

Objectives

The aim of this study was to assess the accuracy of preoperative templating in THR patients, and to assess the difference between templating performed with and without the KingMark device.

Methods​

Our retrospective study included 642 consecutive patients who had primary THR at the Royal Bournemouth Hospital in the UK. Four hundred fifty-three (71%) of patients had the KingMark device on their templated radiographs. Patients who had hybrid total hip replacements using an uncemented acetabular component and cemented femoral component were included in the study. Digital templating was done using TraumaCad software (Brainlab). Analysis of the accuracy of predicting component size has been evaluated by comparing preoperative planned sizes with implanted sizes as documented by the surgeons and labels attached to the operative note. ​

​Results​

The templated size corresponded to the actual femoral implant used in approximately 65.2% of cases. When femoral prostheses within one size above or below the templated size were included,​the accuracy of preoperative templating rose to 97.2%. Regarding the uncemented acetabular component, the templated size corresponded to the actual acetabular implant used in 46.3% of cases. When acetabular cup within one size above or below the templated size were included, the accuracy of preoperative templating rose to 87.5%. Similarly, there was minimal difference between the predicted templated sizes using the KingMark device compared to templating performed without it.

​Conclusions​

Preoperative templating is an essential part in optimizing the outcome of THRs. Templating allows the surgeon to estimate the size of the components to be used. It also provides a starting point, from which the surgeon can proceed from, and saves valuable intraoperative time by assessing the level of the femoral neck osteotomy and the degree of lateral rasping. Multiple factors affect the accuracy of preoperative templating including the patient BMI, external rotation of the hip and surgeon’s experience. Although there are different methods of templating, digital templating with 2D radiographs is likely the most cost-effective and efficient process available at this time.

## Introduction

Elective total hip replacements (THRs) are an established treatment for end-stage pathology of the hip. More than 100,000 THRs were performed in England and Wales during 2020 [1], which is likely to increase in number with an ageing population. Dislocation and instability post-surgery are potential complications and must be avoided [2]. Implants should be selected carefully and positioned correctly, in order to restore centre of rotation and leg length, which can be achieved with preoperative templating. This has been shown to improve postoperative range of motion, stability, reduce operative time, and reduce implant mal-positioning [2]. Traditional templating methods relied on acetate sheets that were superimposed onto plain radiographs [3]. With the advent of digital radiographs, techniques have evolved, and these sheets have largely now been superseded by digital templating software.

The goals of THR templating are to predict femoral and acetabular implant size, assess leg length and offset, and predict implant positioning [4]. Digital templating can be performed using 2D or 3D imaging. It relies on accurate calibration, which can be performed using a marker ball of 25-50mm in diameter, or the application of a KingMark templating device (Brainlab, Munich, Germany).

The aim of this study was to assess the accuracy of digital templating in cemented THRs using the TraumaCad software (Brainlab).

## Materials and methods

**Figure 1:**
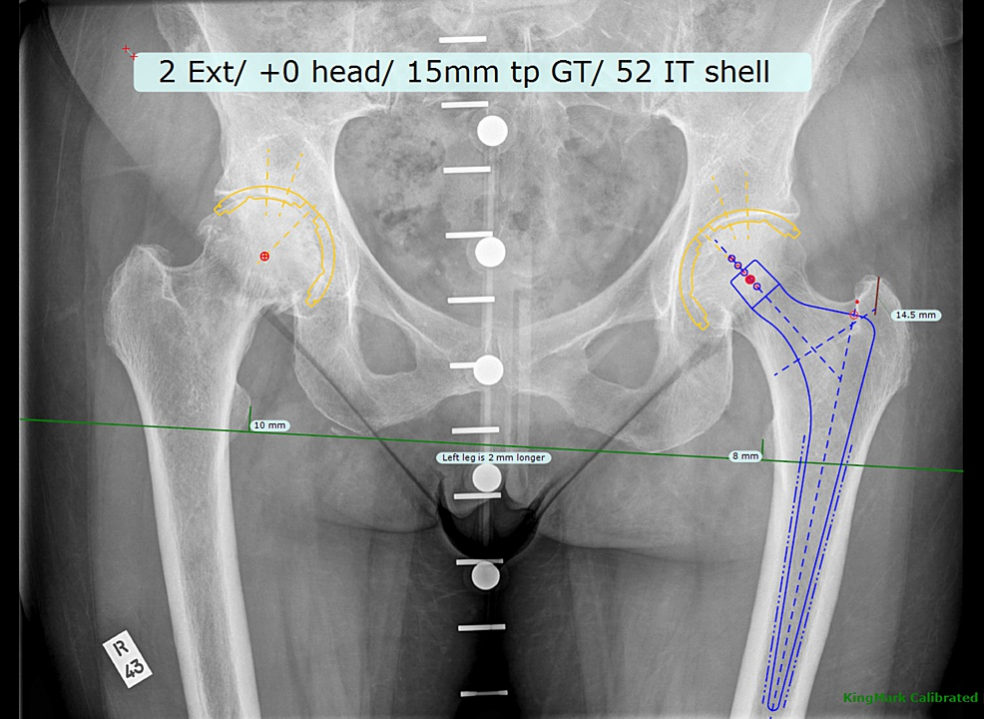
Example of templated hip using TraumaCad software

A retrospective evaluation of patients undergoing THRs at the Royal Bournemouth Hospital between May 2018-December 2019 was performed. Patients were included if they underwent a cemented femoral component with an uncemented acetabulum. Data from multiple surgeons were collected, with all cases performed via a posterior approach to the hip. Primary hybrid THRs are templated prior to surgery using the TraumaCad software and uploaded onto the hospital Picture Archiving Communication System (PACS) (Figure 1). 

The medical notes and digital system were reviewed, with the templated predicted sizes and the actual implant size recorded. Data were entered into an Excel spreadsheet (Microsoft, Redmond, WA, USA), and statistical analysis via frequencies and most common implant size was performed using SPSS version 26 (IBM Corp., Armonk, NY, USA).

Templating

Radiographs were centred onto the pubic symphysis with optimal alignment obtained if the obturator foramen were symmetrical. Anteroposterior (AP) radiographs were then uploaded onto the TraumaCad software. The templating process starts with establishing the centre of rotation, which is in the middle of the acetabular cavity. The centre of rotation is attempted to be met by inserting a femoral component with the option of using a plus or minus attachment. This is provided that the femoral component will fit in the femoral canal with enough space for cement mantle. The templating was performed by the surgeon who was performing the case.

## Results

Between July 2018 to December 2019 the data from 641 THRs were identified. Four hundred one (63%) patients were female, and 240 (37%) were male. All patients underwent hybrid THRs with cemented femoral stems and uncemented acetabular components. The most common acetabular and femoral components inserted for males was a 58 cup with a size 2 stem. The most common acetabular and femoral components inserted for females was a 52 cup with a size 1 stem.

In terms of templating marker, 453 patients (71%) had a KingMark templating device, eight (1%) had a templating ball and 179 (28%) had no templating marker.

The templated size corresponded to the actual femoral implant used in 65.2% of cases (Table 1). When femoral prostheses within one size above or below the templated size were included,​the accuracy of preoperative templating rose to 97.2%, and 100% for within two sizes. Regarding the uncemented acetabular component, the templated size corresponded to the actual acetabular implant used in 46.3% of cases. When acetabular cup within one size above or below the templated size were included, the accuracy of preoperative templating rose to 87.5%, and to 98% for within two sizes.

**Table 1:** Summary of templating accuracy performed for both the femoral and acetabular component

All patients						
Femoral component				Acetabular component		
Predicted size	Frequency	Percentage accuracy	Cumulative Percent	Frequency	Percentage accuracy	Cumulative Percent
Exact	418	65.2	65.2	297	46.3	46.3
+/-1	205	32	97.2	264	41.2	87.5
+/-2	18	2.8	100	67	10.5	98
+/-3	0	0		13	2	100
Total	641	100		641	100	

Templating device

The accuracy of templating both the femoral and acetabular components that had a KingMark templating device are summarised in Tables 2, 3.

**Table 2:** Summary of templating accuracy performed for the femoral component in patients who had the KingMark device and in those without it.

KingMark only templating device						
Femoral component King Mark templating				Femoral component no radiographic marker		
Predicted size	Frequency	Percentage accuracy	Cumulative Percent	Frequency	Percentage accuracy	Cumulative Percent
Exact	296	65.3	65.3	121	67.2	67.2
+/-1	145	32	97.4	55	30.6	97.8
+/-2	12	2.6	100	4	2.2	100
Total	453	100				

**Table 3:** Summary of templating accuracy performed for the acetabular component in patients who had the KingMark device and in those without it.

Acetabular component KingMark templating				Acetabular component no radiographic marker		
Predicted size	Accuracy	Percentage accuracy	Cumulative Percent	Frequency	Percentage accuracy	Cumulative Percent
Exact	213	47	47	84	46.7	46.7
+/-1	183	40.4	87.4	74	41.1	87.8
+/-2	46	10.2	97.6	19	10.6	98.3
+/-3	11	2.4	100	3	1.7	100
Total	453	100		180	100	

## Discussion

There are many advantages of preoperative templating in THRs. Firstly, from a patient safety viewpoint, it ensures that incorrect implants are not inserted. From an economical perspective, templating can reduce the inventory of implants required and reduce costs [3,5]. Additionally, preoperative templating is evidence of surgical planning and preparation prior to a case, which is an advantage in medicolegal scenarios [6].

The results of this study show the use of digital preoperative templating with the TraumaCad software on hybrid CPT stems (Zimmer, Warsaw, IN, USA) and Trilogy acetabular cups (Zimmer) is highly effective at predicting the size of components required for THRs. There was a 65% accuracy for the femoral component, which rose to 97.2% for implants within one size of predicted. Similarly, for the acetabular components, the accuracy was 46.3% which rose to 87.5% for implants within one size of predicted.

There are different methods of preoperative templating for THRs, acetate sheets on digital radiographs, 2D templating using computer software, and 3D templating which relies on cross-sectional imaging such as CT scans of the pelvis [7,8]. A recent meta-analysis suggested that 3D templating may be more accurate than traditional 2D methods, however the clinical benefits of this increased accuracy are not known [9]. Similarly, the costs and radiation exposure of a CT scan compared to a weight-bearing AP hip X-ray, make 3D templating less favourable. 

Templating markers are commonly used in AP pelvic X-rays for calibration purposes. The KingMark device is a double calibration device, which uses markers placed anteriorly and posteriorly to the pelvis [10]. KingMark templating has been shown to improve the accuracy in predicting components in THR surgery compared to a single marker template [11,12]. However, based on our study findings, there was no significant difference in accuracy when using the KingMark template compared to the use of no templating marker. Additionally, a recent study of acetabular componentry templating without markers and a standard calibration of 118%/119% found a 61.1% accuracy within one size, and 96.3% for within two sizes for acetabular components [11]. This could suggest that with the use of the TraumaCad software the KingMark device is not essential for accurate templating.

In comparison to other studies, our predicted templated sizes were more accurate. A recent study of 632 patients had an accuracy of 42% for femoral components and 37% for acetabular components, which rose to 87% and 78% respectively within one size, using the syngo-EndoMap software (Siemens Medical Solutions AG, Erlangen, Germany) [13]. Similarly, a recent study of 391 patients had an accuracy of 27.2% for femoral components and 28.9% for acetabular components, which rose to 61.0% and 63.9%, respectively, using the mediCAD software (mediCAD Hectec GmbH, Altdorf, Germany) [14]. However, the cases in our study have all been hybrid THRs with cemented femoral components. There is some evidence that cemented implant templating may be more accurate than uncemented stems [15,16]. Additionally, this study reports the templating of a single type of acetabular and femoral component, which may have increased the accuracy of the process as familiarity increases.

Limitations of this study include the retrospective nature of this single site study, as well as further demographical data such as BMI and comorbidities were not present. Diagnosis and reason for THRs were not recorded, nor was seniority of the surgeon performing the templating.

## Conclusions

Our study showed that templating matched the true implanted components in a high percentage of the femoral implant at 65%, while the acetabular component matched in 46.3%, when the templated components were within one size accuracy increased to 97.2% in the femoral component and 87.5% in the acetabular component, therefore we recommend templating for all cases as it allows the surgeon to estimate the size of the components to be used. It also provides a starting point from which the surgeon can proceed from and saves valuable intraoperative time by assessing the level of the femoral neck osteotomy and the degree of lateral rasping. Multiple factors affect the accuracy of preoperative templating including the patient BMI, external rotation of the hip and surgeon’s experience. Although there are different methods of templating, digital templating with 2D radiographs is likely the most cost-effective and efficient process available at this time.
